# Health Literacy Recommendations for Digital Mental Health Treatments

**DOI:** 10.3928/24748307-20250714-01

**Published:** 2026-01

**Authors:** Alana C. Fisher, Atria Rezwan, Danielle M. Muscat, Julie Ayre, Madelyne Bisby, Taylor Hathway, Isabella Choi, Nickolai Titov, Blake F. Dear

**Affiliations:** a School of Psychological Sciences, eCentreClinic, Sydney, New South Wales, Australia.; b MQ Health, MindSpot, Macquarie University, Sydney, New South Wales, Australia.; c Sydney School of Public Health, Sydney Health Literacy Lab, Sydney, New South Wales, Australia.; d Faculty of Medicine and Health, Sydney, New South Wales, Australia.; e Sydney Medical School, The University of Sydney, Sydney, New South Wales, Australia.

## Abstract

**Background:**

Digital mental health (dMH) treatments have garnered much attention for increasing access to treatment, yet real-world engagement with these treatments remains a challenge. In upscaling these treatments, we need to ensure that they are equitable and do not exclude groups who already experience inequities in health care, such as people with lower health literacy.

**Objective:**

To co-develop recommendations for the design and delivery of dMH treatments for people with a variety of health literacy levels.

**Methods:**

Drafted recommendations were based on a thematic analysis of 357 free-text comments (likes, dislikes/other suggestions) from 213 people (*n* = 80 lower, and *n* = 133 higher health literacy) who had completed unguided internet-delivered cognitive behaviour therapy (iCBT) for depression and anxiety, as part of a trial. The initial set of drafted recommendations were iteratively modified and refined based on a review by a multidisciplinary project team with professional and lived-experience expertise (*n* = 9) and focus group consultations with people with relevant lived experience (*n* = 8).

**Key Results:**

The co-development process resulted in seven final recommendations: (1) Focus on informative and practical content; (2) Prioritize accessibility and ease of use; (3) Structure content in a progressive, layered way; (4) Enhance interactivity and engagement; (5) Employ strategies to enhance motivation and accountability; (6) Consider participants' emotional wellbeing; (7) Incorporate diverse modes of delivering content. Most recommendations were based on comments from people with lower and higher health literacy.

**Conclusions:**

These recommendations advance both research and practice by outlining a flexible and practical framework for dMH treatment developers and service providers to meet the preferences and needs of people with diverse health literacy strengths and needs. Further research is needed to determine the feasibility and impact of implementing these recommendations across different dMH treatment delivery formats, settings, and populations.

## Abstract (plain language summary)

**Plain Language Summary: **This study developed seven recommendations to improve digital mental health treatments for people with different health literacy levels. These recommendations are based on feedback from users and experts and aim to help make digital treatments easy to access, use, and engage with. These recommendations will also guide people who develop digital treatments, but they need to be tested more.

Digital mental health (dMH) treatments, such as internet-delivered cognitive behavioral therapy (iCBT) programs (Andersson & Titov, 2014) are structured psychological interventions delivered via an internet-connected device (e.g., laptop/desktop or smartphone/tablet). They provide evidence-based information and skills, with or without the guidance of a health professional to reduce symptoms of mental health conditions (Bassilios, Ftanou, et al., 2022; Bassilios, Morgan, et al., 2022), and are offered as part of routine care in several countries around the world (Hadjistavropoulos et al., 2021; Johansson et al., 2019; Nordgreen et al., 2019; Ruwaard et al., 2012; Titov et al., 2020). With at least one-third to one-half of adults in high income countries reporting limited health literacy (Australian Bureau of Statistics, 2006; Baccolini et al., 2021; Kutner, 2006; Sørensen et al., 2015), this group likely represents a significant proportion of potential dMH users.

Health literacy can be defined as “the personal competencies and organizational structures, resources and commitment which enable people to access, understand, appraise and use information and services in ways which promote and maintain good health” (Nutbeam & Muscat, 2023, p. 3). Groups found to experience greater difficulties with health literacy at the individual level include those who speak a main a language other than English (in countries where the main language is English), were born overseas, have lower educational attainment, and are without private health insurance (Beauchamp et al., 2015; Nutbeam & Muscat, 2021). As is the case in other areas of health care (Berkman et al., 2011; Dahl & Hosler, 2020; Degan et al., 2021), people with health literacy difficulties may have poorer access, uptake, and engagement with dMH treatments. As such, some dMH treatments may inadvertently compound the health care inequities that this group already face, and that these treatments seek to overcome. Against this background, there is a need for frameworks and guidelines to inform the design and delivery of dMH treatments, which can accommodate diverse levels of health literacy among prospective users (Cheng et al., 2020).

The need for such frameworks and guidelines may be especially pressing in the context of unguided or self-guided dMH treatments. This is because, unlike their guided counterparts, unguided treatments are completed independently, or with limited technical and administrative support (and risk/safety monitoring) by trained non-clinical personnel, such as a research officer or other clinic support staff (e.g., an automated self-help program or app) (Bassilios, Ftanou, et al., 2022; Bassilios, Morgan, et al., 2022). In addition, people tend to self-refer to these treatments via a direct-to-consumer service delivery module meaning that, in absence of health professional support or guidance, the treatments themselves (i.e., their design, content, format, and delivery mode) need to be accessible from a health literacy perspective. It is also for these reasons, that unguided dMH treatments have attracted considerable interest due to their high potential for scalability and cost-effectiveness. This is particularly the case in settings where challenges in providing universally accessible, affordable, high-quality mental health care and health literacy tend to co-exist (Meherali et al., 2020). While both efficacious (Dear et al., 2015; Titov et al., 2015) and effective (Morgan et al., 2017) at reducing symptoms of depression and anxiety, unguided dMH treatments are associated with poorer adherence, engagement, and symptom improvement than their therapist-guided counterparts (Krieger et al., 2023) Because of this, we need to consider how best to design and deliver dMH treatments, particularly those without guidance from a health professional, to maximize their suitability for people with diverse health literacy strengths and needs.

## Methods

These recommendations were co-developed through a multi-stage linear process involving (1) a multidisciplinary project team of academic researchers (ACF, DMM, JA, MB, NT, BFD), research psychologists (MB, TH, IC, NT, BFD), a lived experience researcher (AR) who used their first-hand experience of mental health service access and health literacy difficulties to provide input as an expert-by-experience (as opposed to by formal training or qualifications); and (2) people with higher and lower health literacy levels who had completed an unguided dMH treatment for anxiety and depression, as part of a research trial of transdiagnostic iCBT addressing both these conditions together (Fisher et al., 2025). Treatment comprised five CBT-based modules or “lessons” delivered over 8 weeks, and each module presented information and skills for managing depression and anxiety, along with practical exercises and additional resources to help participants apply new learnings to their own situation. For this trial, people had self-referred to treatment in response to independent internet searches via social media advertisements (for full methods see: Fisher et al., 2025). Ethics approval for all aspects of the current development process was sought and obtained through Macquarie University Human Research Ethics Committee (reference number: 520211080734189). All participants provided written consent for their data to be used in research.

The development process was as follows:

1. A set of recommendations were drafted by the first author (ACF) based on a thematic analysis of 357 free-text comments from the 213 trial completers (of whom, *n* = 80 lower health literacy; *n* = 133 higher health literacy; **Table A**) (Fisher et al., 2025). Specifically, upon completing the unguided dMH treatment, trial participants completed a post-treatment satisfaction questionnaire which asked them comment on the treatment (referred to as “the course”): “what did you NOT LIKE about the course?”, “What did you LIKE about the course?”, and “…do you have any other feedback or suggestions you would like to share with us?” During thematic analysis, it was noted if the identified themes and subthemes were present in the comments of participants with lower versus higher health literacy (H = higher, L = lower, in the Results). Participants with lower health literacy were those whose mean score at baseline (i.e., initial assessment) corresponded to reporting difficulties on at least one domain of the Health Literacy Questionnaire (HLQ; Australian Bureau of Statistics, 2018; Osborne et al., 2013). Accordingly, means of ≤2 of 4 on HLQ domains 1 to 5 (i.e., corresponding to strongly disagree/disagree), and ≤3 of 5 on HLQ domains 6 to 9 (i.e., corresponding to sometimes/usually/always difficult or cannot do) were taken as having health literacy difficulties (for more details see Fisher et al., 2025).
2. The resulting set of drafted recommendations was then reviewed by the project team, whose expertise covered: clinical psychology, health literacy, dMH treatments, interventions for culturally and linguistically diverse populations, and lived experience of mental health and health literacy difficulties. Drafted recommendations were subsequently iterated via a collaborative working document (hosted on Microsoft Sharepoint) in line with the broader research evidence and other relevant guidelines.
3. Two focus group consultations (co-facilitated by ACF and lived experience researcher, AR) comprised a purposive sample of the original trial participants identified as having lower health literacy (*n* = 8), provided further feedback and suggestions for refining the recommendations. Two focus groups were conducted to accommodate participant availability; discussion topics focused on the perceived relevance, comprehensiveness, and comprehensibility of the recommendations from a lived experience perspective. The focus group consultations did not aim to reach consensus from a lived experience perspective but rather to elicit points of agreement and/or disagreement and ensure that the recommendations reflected a range of views and experiences.
4. Based on the focus group consultations, the recommendations were further revised and refined after a second review by the project team. Key revisions based on consultation feedback were changes to wording to increase clarity, adding in illustrative examples for some of the recommendations (e.g., features that ex-emplify “user-friendly” design) and establishing the extent of agreement for certain recommendations (e.g., “mobile friendly” versus “mobile first” design, inclusion of peers).

**Table A.**
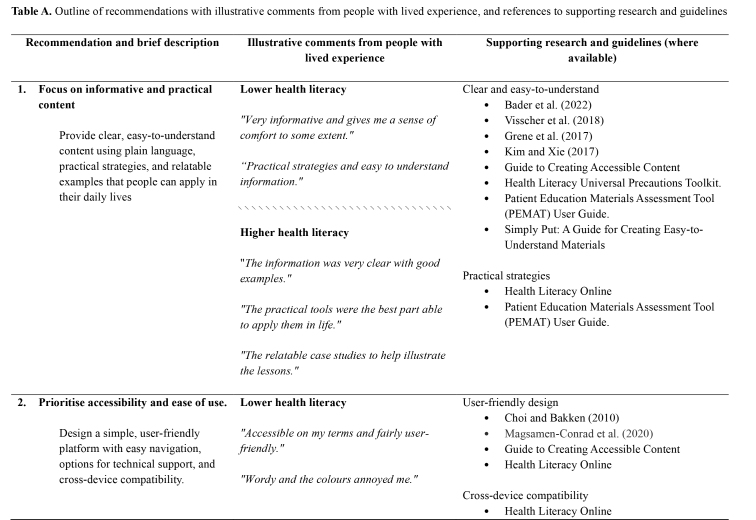
Outline of recommendations with illustrative comments from people with lived experience, and references to supporting research and guidelines

**Recommendation and brief description**	**Illustrative comments from people with lived experience**	**Supporting research and guidelines (where available)**
**1. Focus on informative and practical content**	**Lower health literacy**	Clear and easy-to-understand Bader et al. (2022) Visscher et al. (2018) Grene et al. (2017) Kim and Xie (2017) Guide to Creating Accessible Content Health Literacy Universal Precautions Toolkit. Patient Education Materials Assessment Tool (PEMAT) User Guide. Simply Put: A Guide for Creating Easy-to-Understand Materials
Provide clear, easy-to-understand content using plain language, practical strategies, and relatable examples that people can apply in their daily lives	*“Very informative and gives me a sense of comfort to some extent.”*	
	*“Practical strategies and easy to understand information.”*	
	\\\\\\\\\\\\\\\\\\\\\\\\\\\\\\\	
	**Higher health literacy**	
	“*The information was very clear with good examples.”*	
		Practical strategies Health Literacy Online Patient Education Materials Assessment Tool (PEMAT) User Guide.
	*“The practical tools were the best part able to apply them in life.”*	
	*“The relatable case studies to help illustrate the lessons.”*	

**2. Prioritise accessibility and ease of use.**	**Lower health literacy**	User-friendly design Choi and Bakken (2010) Magsamen-Conrad et al. (2020) Guide to Creating Accessible Content Health Literacy Online
Design a simple, user-friendly platform with easy navigation, options for technical support, and cross-device compatibility.	*“Accessible on my terms and fairly user-friendly.”*	
	*“Wordy and the colours annoyed me.”*	Cross-device compatibility
		Health Literacy Online

**Table:**
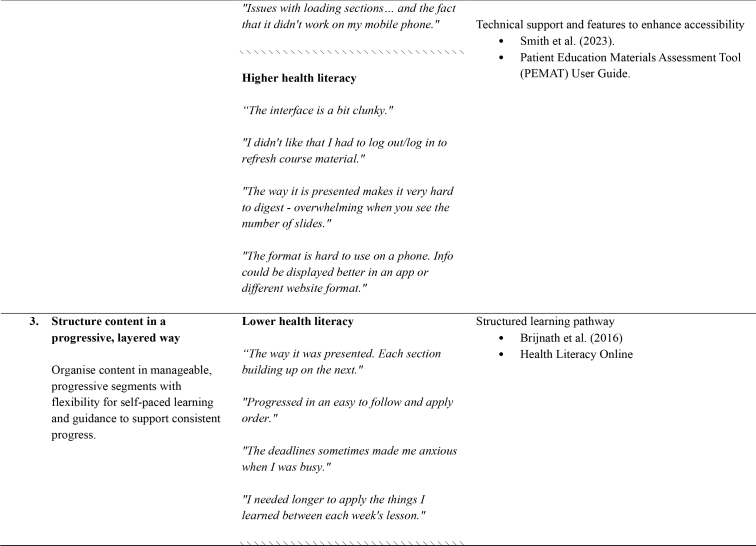


	*“Issues with loading sections… and the fact that it didn't work on my mobile phone.”*	Technical support and features to enhance accessibility Smith et al. (2023). Patient Education Materials Assessment Tool (PEMAT) User Guide.
	\\\\\\\\\\\\\\\\\\\\\\\\\\\\\\\	
	**Higher health literacy**	
	*“The interface is a bit clunky.”*	
	*“I didn't like that I had to log out/log in to refresh course material.”*	
	*“The way it is presented makes it very hard to digest - overwhelming when you see the number of slides.”*	
	*“The format is hard to use on a phone. Info could be displayed better in an app or different website format.”*	

**3. Structure content in a progressive, layered way**	**Lower health literacy**	Structured learning pathway Brijnath et al. (2016) Health Literacy Online
	*“The way it was presented. Each section building up on the next.”*	
Organise content in manageable, progressive segments with flexibility for self-paced learning and guidance to support consistent progress.		
	*“Progressed in an easy to follow and apply order.”*	
	*“The deadlines sometimes made me anxious when I was busy.”*	
	*“I needed longer to apply the things I learned between each week's lesson.”*	
	\\\\\\\\\\\\\\\\\\\\\\\\\\\\\\\

**Table:**
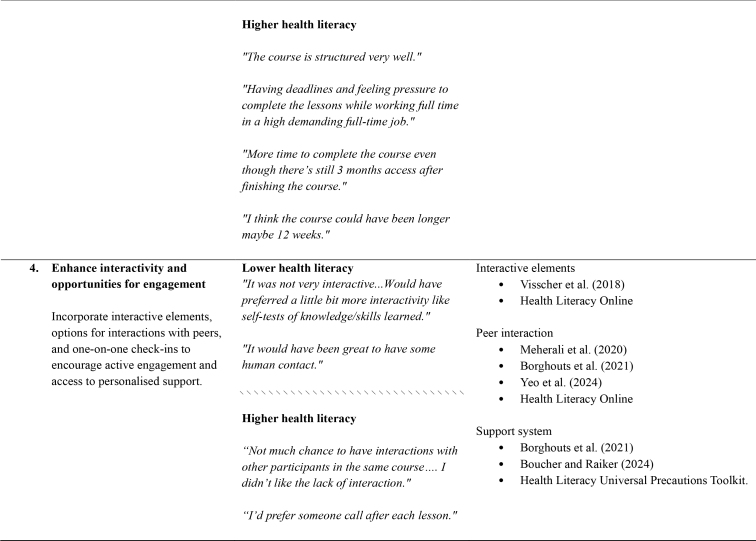


	**Higher health literacy**	
	*“The course is structured very well.”*	
	*“Having deadlines and feeling pressure to complete the lessons while working full time in a high demanding full-time job.”*	
	*“More time to complete the course even though there's still 3 months access after finishing the course.”*	
	*“I think the course could have been longer maybe 12 weeks.”*	

**4. Enhance interactivity and opportunities for engagement**	**Lower health literacy**	Interactive elements Visscher et al. (2018) Health Literacy Online
	*“It was not very interactive...Would have preferred a little bit more interactivity like self-tests of knowledge/skills learned.”*	
Incorporate interactive elements, options for interactions with peers, and one-on-one check-ins to encourage active engagement and access to personalised support.		
		Peer interaction Meherali et al. (2020) Borghouts et al. (2021) Yeo et al. (2024) Health Literacy Online
	*“It would have been great to have some human contact.”*	
	\\\\\\\\\\\\\\\\\\\\\\\\\\\\\\\	
	**Higher health literacy**	
		Support system Borghouts et al. (2021) Boucher and Raiker (2024) Health Literacy Universal Precautions Toolkit.
	*“Not much chance to have interactions with other participants in the same course…. I didn't like the lack of interaction.”*	
	*“I'd prefer someone call after each lesson.”*

**Table:**
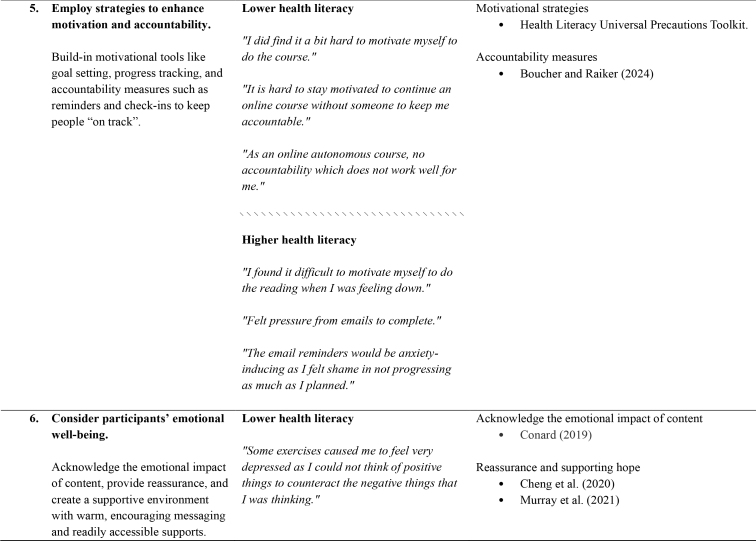


**5. Employ strategies to enhance motivation and accountability.**	**Lower health literacy**	Motivational strategies Health Literacy Universal Precautions Toolkit.
	*“I did find it a bit hard to motivate myself to do the course.”*	
Build-in motivational tools like goal setting, progress tracking, and accountability measures such as reminders and check-ins to keep people “on track”.		Accountability measures Boucher and Raiker (2024)
	*“It is hard to stay motivated to continue an online course without someone to keep me accountable.”*	
	*“As an online autonomous course, no accountability which does not work well for me.”*	
	\\\\\\\\\\\\\\\\\\\\\\\\\\\\\\\	
	**Higher health literacy**	
	*“I found it difficult to motivate myself to do the reading when I was feeling down.”*	
	*“Felt pressure from emails to complete.”*	
	*“The email reminders would be anxiety-inducing as I felt shame in not progressing as much as I planned.”*	

**6. Consider participants' emotional well-being.**	**Lower health literacy**	Acknowledge the emotional impact of content Conard (2019)
	*“Some exercises caused me to feel very depressed as I could not think of positive things to counteract the negative things that I was thinking.”*	
Acknowledge the emotional impact of content, provide reassurance, and create a supportive environment with warm, encouraging messaging and readily accessible supports.		Reassurance and supporting hope Cheng et al. (2020) Murray et al. (2021)

**Table:**
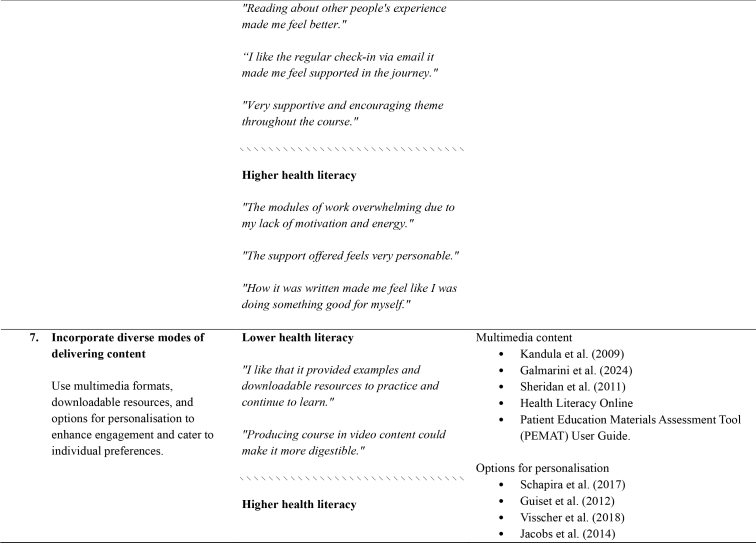


	*“Reading about other people's experience made me feel better.”*	
	*“I like the regular check-in via email it made me feel supported in the journey.”*	
	*“Very supportive and encouraging theme throughout the course.”*	
	\\\\\\\\\\\\\\\\\\\\\\\\\\\\\\\	
	**Higher health literacy**	
	*“The modules of work overwhelming due to my lack of motivation and energy.”*	
	*“The support offered feels very personable.”*	
	*“How it was written made me feel like I was doing something good for myself.”*	

**7. Incorporate diverse modes of delivering content**	**Lower health literacy**	Multimedia content Kandula et al. (2009) Galmarini et al. (2024) Sheridan et al. (2011) Health Literacy Online Patient Education Materials Assessment Tool (PEMAT) User Guide.
	*“I like that it provided examples and downloadable resources to practice and continue to learn.”*	
Use multimedia formats, downloadable resources, and options for personalisation to enhance engagement and cater to individual preferences.		
	*“Producing course in video content could make it more digestible.”*	
		Options for personalisation Schapira et al. (2017) Guiset et al. (2012) Visscher et al. (2018) Jacobs et al. (2014)
	\\\\\\\\\\\\\\\\\\\\\\\\\\\\\\\	
	**Higher health literacy**

**Table:**
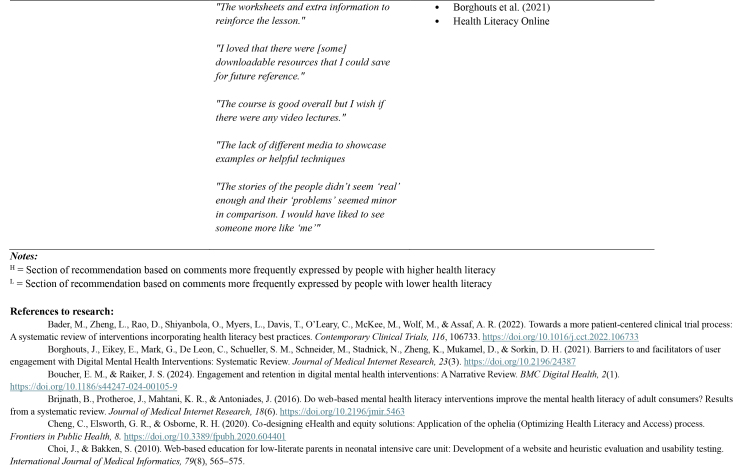

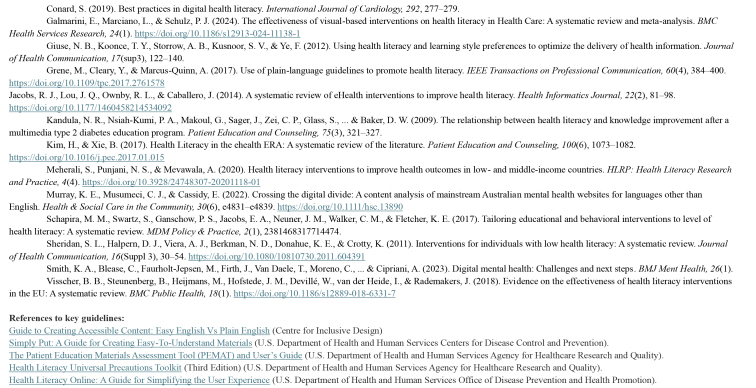


	*“The worksheets and extra information to reinforce the lesson.”*	Borghouts et al. (2021) Health Literacy Online
	*“I loved that there were [some] downloadable resources that I could save for future reference.”*	
	*“The course is good overall but I wish if there were any video lectures.”*	
	*“The lack of different media to showcase examples or helpful techniques*	
	*“The stories of the people didn't seem ‘real’ enough and their ‘problems’ seemed minor in comparison. I would have liked to see someone more like ‘me’”*	

***Notes:***

H = Section of recommendation based on comments more frequently expressed by people with higher health literacy

L = Section of recommendation based on comments more frequently expressed by people with lower health literacy

**References to research:**

Bader, M., Zheng, L., Rao, D., Shiyanbola, O., Myers, L., Davis, T., O'Leary, C., McKee, M., Wolf, M., & Assaf, A. R. (2022). Towards a more patient-centered clinical trial process: A systematic review of interventions incorporating health literacy best practices. *Contemporary Clinical Trials, 116*, 106733.https://doi.org/10.1016/j.cct.2022.106733

Borghouts, J., Eikey, E., Mark, G., De Leon, C., Schueller, S. M., Schneider, M., Stadnick, N., Zheng, K., Mukamel, D., & Sorkin, D. H. (2021). Barriers to and facilitators of user engagement with Digital Mental Health Interventions: Systematic Review. *Journal of Medical Internet Research, 23*(3).https://doi.org/10.2196/24387

Boucher, E. M., & Raiker, J. S. (2024). Engagement and retention in digital mental health interventions: A Narrative Review. *BMC Digital Health, 2*(1). https://doi.org/10.1186/s44247-024-00105-9

Brijnath, B., Protheroe, J., Mahtani, K. R., & Antoniades, J. (2016). Do web-based mental health literacy interventions improve the mental health literacy of adult consumers? Results from a systematic review. *Journal of Medical Internet Research, 18*(6).https://doi.org/10.2196/jmir.5463

Cheng, C., Elsworth, G. R., & Osborne, R. H. (2020). Co-designing eHealth and equity solutions: Application of the ophelia (Optimizing Health Literacy and Access) process. *Frontiers in Public Health, 8.*https://doi.org/10.3389/fpubh.2020.604401

Choi, J., & Bakken, S. (2010). Web-based education for low-literate parents in neonatal intensive care unit: Development of a website and heuristic evaluation and usability testing. *International Journal of Medical Informatics, 79*(8), 565–575.

Conard, S. (2019). Best practices in digital health literacy. *International Journal of Cardiology, 292*, 277–279.

Galmarini, E., Marciano, L., & Schulz, P. J. (2024). The effectiveness of visual-based interventions on health literacy in Health Care: A systematic review and meta-analysis. *BMC Health Services Research, 24*(1).https://doi.org/10.1186/s12913-024-11138-1

Giuse, N. B., Koonce, T. Y., Storrow, A. B., Kusnoor, S. V., & Ye, F. (2012). Using health literacy and learning style preferences to optimize the delivery of health information. *Journal of Health Communication, 17*(sup3), 122–140.

Grene, M., Cleary, Y., & Marcus-Quinn, A. (2017). Use of plain-language guidelines to promote health literacy. *IEEE Transactions on Professional Communication, 60*(4), 384–400. https://doi.org/10.1109/tpc.2017.2761578

Jacobs, R. J., Lou, J. Q., Ownby, R. L., & Caballero, J. (2014). A systematic review of eHealth interventions to improve health literacy. *Health Informatics Journal, 22*(2), 81–98. https://doi.org/10.1177/1460458214534092

Kandula, N. R., Nsiah-Kumi, P. A., Makoul, G., Sager, J., Zei, C. P., Glass, S., ... & Baker, D. W. (2009). The relationship between health literacy and knowledge improvement after a multimedia type 2 diabetes education program. *Patient Education and Counseling, 75*(3), 321–327.

Kim, H., & Xie, B. (2017). Health Literacy in the ehealth ERA: A systematic review of the literature. *Patient Education and Counseling, 100*(6), 1073–1082.https://doi.org/10.1016/j.pec.2017.01.015

Meherali, S., Punjani, N. S., & Mevawala, A. (2020). Health literacy interventions to improve health outcomes in low- and middle-income countries. *HLRP: Health Literacy Research and Practice, 4*(4).https://doi.org/10.3928/24748307-20201118-01

Murray, K. E., Musumeci, C. J., & Cassidy, E. (2022). Crossing the digital divide: A content analysis of mainstream Australian mental health websites for languages other than English. *Health & Social Care in the Community, 30*(6), e4831–e4839.https://doi.org/10.1111/hsc.13890

Schapira, M. M., Swartz, S., Ganschow, P. S., Jacobs, E. A., Neuner, J. M., Walker, C. M., & Fletcher, K. E. (2017). Tailoring educational and behavioral interventions to level of health literacy: A systematic review. *MDM Policy & Practice, 2*(1), 2381468317714474.

Sheridan, S. L., Halpern, D. J., Viera, A. J., Berkman, N. D., Donahue, K. E., & Crotty, K. (2011). Interventions for individuals with low health literacy: A systematic review. *Journal of Health Communication, 16*(Suppl 3), 30–54.https://doi.org/10.1080/10810730.2011.604391

Smith, K. A., Blease, C., Faurholt-Jepsen, M., Firth, J., Van Daele, T., Moreno, C., ... & Cipriani, A. (2023). Digital mental health: Challenges and next steps. *BMJ Ment Health, 26*(1).

Visscher, B. B., Steunenberg, B., Heijmans, M., Hofstede, J. M., Devillé, W., van der Heide, I., & Rademakers, J. (2018). Evidence on the effectiveness of health literacy interventions in the EU: A systematic review. *BMC Public Health, 18*(1).https://doi.org/10.1186/s12889-018-6331-7

**References to key guidelines:**

Guide to Creating Accessible Content: Easy English Vs Plain Englishhttps://centreforinclusivedesign.org.au/wp-content/uploads/2020/04/Easy-English-vs-Plain-English_accessible.pdf (Centre for Inclusive Design)

Simply Put: A Guide for Creating Easy-To-Understand Materialshttps://stacks.cdc.gov/view/cdc/11938 (U.S. Department of Health and Human Services Centers for Disease Control and Prevention).

The Patient Education Materials Assessment Tool (PEMAT) and User's Guidehttps://www.ahrq.gov/health-literacy/patient-education/pemat.html (U.S. Department of Health and Human Services Agency for Healthcare Research and Quality).

https://www.ahrq.gov/sites/default/files/publications2/files/health-literacy-toolkit-third-edition.pdf (Third Edition) (U.S. Department of Health and Human Services Agency for Healthcare Research and Quality).

Health Literacy Online: A Guide for Simplifying the User Experiencehttps://health.gov/healthliteracyonline/ (U.S. Department of Health and Human Services Office of Disease Prevention and Health Promotion).

### Patient and Public Involvement

People with lived experience of mental health concerns and health literacy difficulties were involved in all aspects of the development process as outlined above. Of note, a lived experience researcher (second author, AR) provided substantial input into the initial drafting and refinement of the recommendations and the manuscript, as well as co-facilitation of the focus groups.

## Results

The thematic analysis and co-development process produced seven recommendations, which relate to the structure, design, content, and delivery of dMH treatments. For each of the recommendations, **Table A** provides references to supporting research and guidelines, as well as illustrative quotes and comments from people with lived experience.

### Recommendation 1: Focus on Informative and Practical Content

***Clear and easy-to-understand. ***Ensure the content of the intervention covers the main important points in an easy-to-understand way, using plain language. or “Easy English.” Use examples to illustrate key points and avoid the use of technical language and jargon where possible (L, H).

***Practical strategies.*** Focus on practical strategies and relatable case story examples that participants can easily apply and “action” in their daily lives (L, H).

### Recommendation 2: Prioritize Accessibility and Ease of Use

***User-friendly design. ***Design the digital platform to be user-friendly, with a simple and uncluttered interface and intuitive navigation between the different modules/sections of the platform. The platform should contain minimal tabs/pages, with all core content and resources consolidated into a single area for easy access (L, H).

***Cross-device compatibility.*** Ensure the intervention is accessible on both laptop/desktop computers and mobile devices (e.g., smart phones/tablets). People reported accessing the content across multiple devices, preferencing mobile phones for convenience and laptop/desktop computers for more focused or in-depth work (L, H).

***Technical support.*** Ensure that participants can easily access technical support and/or in-built resources to trouble-shoot any issues, these may include FAQs and tutorial-style navigation videos (L, H).

***Other design features to enhance accessibility. ***Incorporate accessibility features such as adjustable text sizes, contrast settings, white space, and audio options for those with visual impairments or reading difficulties. Videos should be accompanied by closed captions and/or a transcript (L, H).

### Recommendation 3: Structure Content in a Progressive, Layered Way

***Structured learning pathway.*** Ensure the content follows a structured and progressive pathway, layering sections so that each one gradually building upon the previous one. Sections presenting more foundational information may be recommended for people seeking help for the first time. Later sections may remain “locked” until these foundational sections are completed. In each new section, include summaries of the key information and concepts covered in previous sections. Consider support extended or ongoing access to course materials after completion, while participants continue to apply new knowledge, practice skills, and integrate these into their lives (L, H).

***Manageable segments.*** Break down the content into smaller, more manageable segments or “chunks” to avoid information overload and feelings of overwhelm among participants (L).

***Scheduling and time constraints. ***Ensure that the course schedule includes some flexibility to avoid participants feeling “rushed” or unable to keep up. Allow flexible, self-paced learning to accommodate individual schedules. Participants with lower health literacy reported needing more time than offered between lessons to apply newly introduced concepts. To ensure consistent progress through the course, there may be value in guiding participants at the start to schedule in regular times for accessing the course material (L, H).

### Recommendation 4: Enhance Interactivity and Opportunities for Engagement

***Interactive elements. ***Incorporate more interactive elements such as drag-and-drop or tick-the-box activities, to encourage engagement. Interactive quizzes were seen as a helpful tool for tracking progress and checking understanding. Any quizzes should be framed as a “check in,” be presented soon after relevant content and include the option for multiple or unlimited attempts (L, H).

***Peer interaction. ***As appropriate, include opportunities for peer interaction and peer- or other facilitator-led discussions which allow people to “see” newly introduced concepts in their own context. Such opportunities should remain optional and include moderated forums, informal group chats, or live sessions with other participants, peer workers, and/or others (e.g., a professional facilitator) with experience and knowledge of the course (L).

***Support system.*** Provide the option for regular one-toone “check-ins” via email or phone calls with a mental health professional, especially for those participants who need more personalized guidance or support. This option should be clearly signposted in the digital platform and easily accessible (e.g., via a direct link to contact the mental health professional) (L).

### Recommendation 5: Employ Strategies to Enhance Motivation and Accountability

***Motivational strategies.*** Incorporate strategies to help participants stay motivated, such as tools to help with setting goals, scheduling regular time for logging in to review the course, tracking progress, and receiving positive reinforcement. Interactive quizzes were seen as one strategy for tracking progress and enhancing motivation (see Guideline 4: Interactive elements) (L, H).

***Accountability measures.*** Integrate measures to promote accountability and help participants to “stay on track”, such as opting-in to regular check-ins, reminders, and one-on-one support sessions (L).

### Recommendation 6: Consider Participants' Emotional Well-Being

***Acknowledge the emotional impact of content.*** Acknowledge and normalize early on that certain content, such as case studies or working though practical exercises (e.g., exposure activities), may be challenging. Explain that fluctuations in symptoms and emotions are normal, and that improvement is not always steady/setbacks can occur. At the same time, highlight available supports (e.g., in the form of “check ins” or supplementary resources) and providing direct links (see Guideline 4: Support system) (L, H).

***Supportive digital environment. ***Create a supportive digital environment for participants via an encouraging, warm and positive tone, language, and messaging, as well as access to regular check-ins (via automated or tailored emails or via human contact) if available (L, H).

***Reassurance and supporting hope. I***nclude elements that provide reassurance and increase hope, such as relatable patient case stories which cover a diverse range of backgrounds, symptom presentations and life circumstances. Depending on available resources and technical limitations, these case stories may be tailored to increase the “match” with the individual participant. Also balance reassurance with normalizing that therapy often involves experiencing some discomfort (L, H).

### Recommendation 7: Incorporate Diverse Modes of Delivering Content

***Multimedia content.*** Where resources are available, use a variety of media formats such as videos, infographics, and audio clips to make the content more engaging and easy-to-understand. Supplementary hard-copy or downloadable resources to accompany online materials may also be considered. Participants suggested including a greater variety of media, especially video content to introduce and illustrate key concepts (L, H).

***Options for personalization. ***Where resources are available, provide participants with options for personalizing or tailoring the course content and format, such that where participants can choose or “build” a patient case story based on elements that resonate with their personal background and experience of mental health concerns (see Guideline 7: Reassurance and supporting hope). According to participants, allowing flexible course delivery with options to tailor the course to their individual preferences and needs (e.g., video/audio versus written content; one-to-one check-ins versus group chats), it would enhance their engagement with the content. This said, in light of available resources, priority should be given to course delivery options that are broadly applicable and accessible to most people (L, H).

## Discussion

The current co-development work resulted in a set of recommendations for the design and development of dMH treatments to accommodate people with diverse health literacy strengths and needs. Although these recommendations are based on data sourced from an unguided dMH treatment, they have implications for the development of both unguided and guided dMH treatments that are accessible, usable, and engaging. Key recommendations include providing clear, easy-to-understand, and easy-to-navigate information using multimedia, and practical, actionable examples; a structured, yet flexible learning pathway for progressive learning; and opportunities for interacting with both the content itself and with others, within a supportive digital environment.

Of note, some recommendations were based mainly on the views of people with lower levels of health literacy. These recommendations related mainly to providing guidance and support, such as opportunities for interaction with peers, one-to-one “check-ins” with a mental health professional for support, and measures to promote accountability and “staying on track”. While these recommendations were developed in the context of an unguided dMH treatment, they suggest that people with lower health literacy may especially benefit from therapist- or peer-guided dMH treatments. This said, most recommendations reflect the views of people with lower and higher levels of health literacy. As such, a universal precautions approach (Brega et al., 2015) to the design and delivery of dMH treatments may be acceptable and useful in meeting most users' needs and preferences. This approach assumes that any user may experience health literacy difficulties and that designing for those with difficulties can benefit everyone.

Broadly speaking, these recommendations align with extant research and guidelines for producing accessible information, including health information and resources for people with lower health literacy. With regards to the research base, some recommendations with empirical support include using: (1) clear, easy-to-understand language to improve user comprehension (Bader et al., 2022; Grene et al., 2017; Visscher et al., 2018) (2) a combination of multimedia content and visual examples to simplify complex health information and aid understanding (Galmarini et al., 2024); and (3) positive tone and framing in health education materials as ‘best practice’ (Niebaum et al., 2015). By contrast, other recommendations have been examined less in the literature and so still have more limited empirical support; for example, programs integrating peer support to ameliorate challenges with health literacy and reduce health inequities (Harris et al., 2015). In further developing these recommendations for the dMH setting, which were based mainly on free-text survey data sourced from previous trial participants, researchers and other relevant stakeholders may wish to conduct more in-depth qualitative work or employ consensus building methods, such as a Delphi study.

With regards to other key guidelines (Brega et al., 2015; Centers for Disease Control and Prevention U.S. Department of Health and Human Services, 2009; Centre for Inclusive Design, 2021; Shoemaker et al., 2014; U.S. Department of Health and Human Services Office of Disease Prevention and Health Promotion, 2016), the current recommendations both complement and extend upon these guidelines. Specifically, these recommendations consider the preferences, needs, and priorities of people seeking treatment for mental health conditions, a group who has been shown to experience greater health literacy difficulties than the general population or other populations with physical health conditions (Degan et al., 2019, 2021), possibly due to the cognitive and functional impacts of mental health conditions and/or the overlap with other risk factors for lower health literacy (e.g., socioeconomic disadvantage) (Clausen et al., 2016). In addition, these recommendations also recognize the concurrent digital literacy demands that digital technologies introduce and that dMH treatments need to accommodate, as these may interact with users' health literacy difficulties (Cheng et al., 2020; Kozelka et al., 2023; van Kessel et al., 2022).

These recommendations are intended to provide a flexible framework for dMH treatment developers and providers looking to maximize the accessibility and usability of their treatments for people with diverse health literacy levels. While these recommendations incorporate lived experience perspectives, future research is needed to evaluate the applicability of these recommendations in different contexts (e.g., therapist-guided dMH treatments) and populations (i.e., e.g., co-occurring physical health conditions, or more severe mental illness) Future research is also needed to determine the impact of these guidelines, and whether their implementation in the design and delivery of dMH treatments leads improved acceptability, usability, and treatment outcomes among people experiencing health literacy difficulties.

In considering the current recommendations, it is important to note they are intended as a guide and not as a prescriptive set of development requirements. As such, which, how and the extent to which the recommendations are implemented will need to consider the unique clinical and contextual factors for certain interventions, populations, or services. A key part of implementation may be multi-stakeholder discovery sessions involving a needs assessment and/or scoping of requirements and available resources (Andersson et al., 2019; Damschroder et al., 2009, 2022; Powell et al., 2014). For example, not all service settings will have the technological infrastructure or budget to implement every feature in the recommendations (e.g., incorporating interactive elements and flexible modes of content delivery). Additionally, not all recommended features may be suitable for all populations (e.g., people with more severe mental health symptoms, co-occurring cognitive difficulties, youth or older adults), and treatment developers will need to balance accessibility with treatment fidelity and efficacy or effectiveness.

In conclusion, this study described a multi-step process which resulted in seven recommendations for the development of dMH treatments, which may help increase the suitability of these interventions for people with diverse health literacy strengths and needs. These recommendations were co-developed with people with lived experience and clinical experts and although some require further empirical evaluation, most represent a set of practical considerations that are likely to positively promote acceptability, engagement and retention in dMH treatments.
